# Draft Genome Sequence of a Novel *Lactobacillus salivarius* Strain Isolated from Piglet

**DOI:** 10.1128/genomeA.01231-13

**Published:** 2014-02-13

**Authors:** Donald A. MacKenzie, Kirsten McLay, Stefan Roos, Jens Walter, David Swarbreck, Nizar Drou, Lisa C. Crossman, Nathalie Juge

**Affiliations:** a The Gut Health and Food Safety Programme, Institute of Food Research, Norwich Research Park, Norwich, United Kingdom; b The Genome Analysis Centre, Norwich Research Park, Norwich United Kingdom; c Department of Microbiology, Swedish University of Agricultural Sciences, Uppsala, Sweden; d Department of Food Science and Technology, University of Nebraska, Lincoln, Nebraska, USA; e School of Biological Sciences, University of East Anglia, Norwich, United Kingdom

## Abstract

*Lactobacillus salivarius* is part of the vertebrate indigenous microbiota of the gastrointestinal tract, oral cavity, and milk. The properties associated with some *L. salivarius* strains have led to their use as probiotics. Here we describe the draft genome of the pig isolate *L. salivarius* cp400, providing insights into host-niche specialization.

## GENOME ANNOUNCEMENT

The Gram-positive bacterium *Lactobacillus salivarius* is an important member of the pig microbiota (1) and a promising probiotic candidate frequently isolated from human, porcine, and avian gastrointestinal tracts, many of which are producers of unmodified bacteriocins (2). The apparent ability of *L. salivarius* to produce very different bacteriocins using the same cellular machinery suggests an unknown versatility mechanism with respect to the production of this dominant probiotic trait (3).

To gain novel insights into the molecular basis underlying *L. salivarius* adaptation to the intestinal ecological niche, we have determined the genome sequence of the pig isolate *L. salivarius* cp400, isolated from preweaned piglet feces (Stefan Roos, unpublished data). Genomic DNA was purified using a modified method from Oh and colleagues (4) and used to generate over 4.65 Gbp of sequence on the Illumina HiSeq2000 platform. Reads passing default filter settings were assembled using Abyss (5) and generated 89 scaffolds containing 87 large scaffolds (>500 bp) and spanning 2,155,512 bp of sequence. This genome assembly of *L. salivarius* cp400 is 2,156,840 bp in length, with an average G+C content of 33.2%. Automatic gene prediction was performed using Glimmer3 and GeneMark (6, 7). Annotation was transferred from the related strain, *L. salivarius* ATCC 11741. Unique regions were manually annotated using Artemis (8), augmented with InterPro (9), TMHMM (10), and SignalP (11). A total of 2,044 protein-coding sequences was predicted, with a coding percentage of 83.7%. Coding density was 0.947 genes per kb with an average gene length of 883 bp. Phylogenetic analysis of the whole-genome sequence of cp400 with the genome sequences available for *L. salivarius* strains ATCC 11741 (human), CECT 5713 (human), UCC118 (human), GJ-24 (human), NIAS840 (chicken), and SMXD51 (chicken) indicates that cp400 is genetically distinct, falling into a separate phylogenetic clade. Comparison of cp400 with the above strains revealed the presence of a megaplasmid of approximately 0.29 Mb and 31.9% G+C, harboring a bacteriocin gene cluster with flanking transposase genes. The cp400 megaplasmid showed similarity with the plasmids pMP188 from the human strain UC118 and pLS51A from the chicken isolate SMXD51 at 91.9% and 91.3% DNA identity, respectively, for matches of >2,000 bp. Analysis of the bacteriocin gene cluster revealed high homology to the Abp118 gene cluster, encoding a class IIb two-peptide bacteriocin composed of Abp118alpha, exhibiting antimicrobial activity, and Abp118beta, which enhanced the antimicrobial activity in *L. salivarius* UCC118 (12). The cp400 bacteriocin displayed 100% identity to the mature alpha and beta peptides of salivaricin P produced by five other pig intestinal isolates of *L. salivarius* (13), and the cluster also contained genes encoding homologues of AbpIP (inducer), AbpK (sensory transduction histidine kinase), AbpR (response regulator), and AbpD (accessory export), suggesting the presence of a functional salivaricin P cluster in the cp400 megaplasmid. There was lower similarity with the salivaricin cluster from human strain CRL1328 (14) at 87.2% DNA identity for matches over 2,000 bp.

Detailed analysis of the assembled cp400 genome will help determine specific genetic features driving genome-wide specialization of *L. salivarius* in response to a particular niche.

### Nucleotide sequence accession numbers.

This genome sequencing project has been deposited at EMBL/DDBJ/GenBank via the European Nucleotide Archive (ENA). The 89 fully annotated scaffolds have been deposited under the accession numbers CBVR010000001 through CBVR010000089.
